# Molecular Pathophysiology of Epithelial Barrier Dysfunction in Inflammatory Bowel Diseases

**DOI:** 10.3390/proteomes6020017

**Published:** 2018-03-31

**Authors:** Jessica Y. Lee, Valerie C. Wasinger, Yunki Y. Yau, Emil Chuang, Vijay Yajnik, Rupert WL. Leong

**Affiliations:** 1 School of Medical Sciences, Faculty of Medicine, The University of New South Wales, Sydney 2052, NSW, Australia; jessyouminlee@gmail.com; 2 Bioanalytical Mass Spectrometry Facility, Mark Wainwright Analytical Centre, The University of New South Wales, Sydney 2052, NSW, Australia; 3 South West Sydney Clinical School, Faculty of Medicine, The University of New South Wales, Sydney 2052, NSW, Australia; yunki.yau@health.nsw.gov.au(Y.Y.Y.); rupert.leong@health.nsw.gov.au (R.W.L.); 4 Department of Gastroenterology, Concord Repatriation General Hospital, Sydney 2139, NSW, Australia; 5 Translational Medicine and Early Clinical, Takeda Pharmaceuticals, Cambridge, MA 02139, USA; Emil.Chuang@takeda.com (E.C.); Vijay.Yajnik@takeda.com (V.Y.); 6 Gastrointestinal Unit, Massachusetts General Hospital, Harvard Medical School, 55 Fruit Street, Boston, MA 02114, USA

**Keywords:** intestinal barrier function, inflammatory bowel disease

## Abstract

Over the years, the scientific community has explored myriads of theories in search of the etiology and a cure for inflammatory bowel disease (IBD). The cumulative evidence has pointed to the key role of the intestinal barrier and the breakdown of these mechanisms in IBD. More and more scientists and clinicians are embracing the concept of the impaired intestinal epithelial barrier and its role in the pathogenesis and natural history of IBD. However, we are missing a key tool that bridges these scientific insights to clinical practice. Our goal is to overcome the limitations in understanding the molecular physiology of intestinal barrier function and develop a clinical tool to assess and quantify it. This review article explores the proteins in the intestinal tissue that are pivotal in regulating intestinal permeability. Understanding the molecular pathophysiology of impaired intestinal barrier function in IBD may lead to the development of a biochemical method of assessing intestinal tissue integrity which will have a significant impact on the development of novel therapies targeting the intestinal mucosa.

## 1. Introduction

The Inflammatory Bowel Diseases (IBD) including ulcerative colitis (UC) and Crohn’s disease (CD) are chronic relapsing disorders of the gastrointestinal tract [1]. The intestinal epithelium is a dynamic ecosystem that maintains a perpetual cycle of death and renewal of the epithelial lining while preserving an elegant balance of immune education, immune response, and immune tolerance to the microorganisms in the intestinal lumen [2]. Evaluation of barrier function in IBD has been shown to reflect disease activity and may have the potential to predict disease course [2,3,4,5,6,7,8,9,10,11,12,13]. However without a standard validated method of intestinal barrier function assessment, it is difficult to compare and compile findings in this important field. Additionally, the exact mechanisms associated with defective barrier functions and IBD remains largely unknown. Better understanding of such phenomenon may unravel an important pathophysiological process of IBD as well as setting a foundation for the development of a biochemical method of assessing and measuring intestinal tissue integrity. This will have significant implications for directing and evaluating future research for novel therapies targeting the intestinal mucosa. This review will examine the components of the epithelial barrier, the pathological changes that result in impaired intestinal permeability and its significance in IBD, and current methods of assessing barrier function. Barrier function is one side of the dichotomy of the host-environment interface, with the balance of these two elements being intrinsic to IBD pathogeneses [14]. The magnitude of the intestinal flora, together with its even more numerous and intricate pathogenic pathways, is a subject matter in itself that requires focused scrutiny. We have thus focused this review on the intestinal epithelium with limited illustrations of significant immunological and microbial interactions when required for context. Recent reviews on the microbial side of the intestinal barrier-environmental interface can be found elsewhere [14,15,16].

## 2. The Intestinal Epithelial Barrier

The intestinal mucosal barrier is a dynamic structure that separates the intestinal lumen and the sterile extracellular internal milieu of the body. Controlled communication between the intestinal lumen and the body is essential for the absorption of nutrients, electrolytes, and water, as well as for the immune system to greet the microbiota and defend against toxins and pathogens [17]. The intestinal barrier consists of the intestinal epithelium, the overlaying mucus layer containing mucin, and various antimicrobial peptides [2]. 

**Figure 1:**
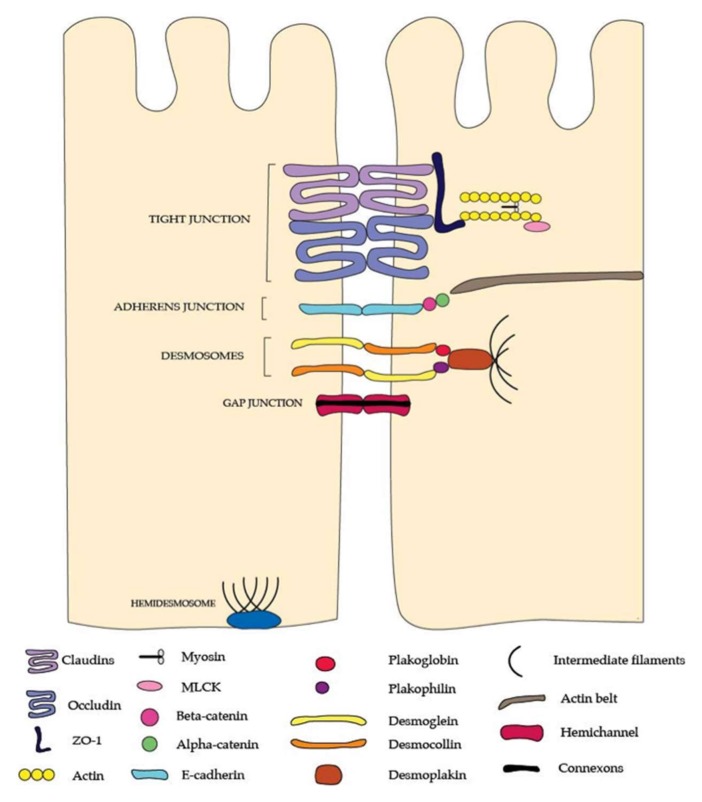
The junctional complexes of the intestinal barrier. Tight junctions are made up of the claudin and occludinmembrane proteins which bind together to seal the paracellular gap between epithelial cells. Zonulin-1 (ZO-1) binds the tight junction complex to the actin cytoskeleton and also regulates the selective passage of macromolecules through the tight junction. The lower junctional complex is the adherens junction which is made up of E-cadherin proteins that attach adjacent epithelial cells. These proteins are anchored by beta-catenin and alpha catenin to the actin cytoskeleton. The deepest junctional complexes at the baselateral end of the epithelial cells are the desmosomes and hemidesmosomes which attach epithelial cells to each other and also to the basement membrane, respectively. Desmosomes are made up of desmoglein and desmocollin partner proteins which are anchored to the filament lattice structure by plakogobin and plakophilin proteins, which in turn attach to desmoplakin. Whilst tight junctions have a primary role in regulating selective ion absorbance from the lumen to the extracellular internal milieu of the body, adherens junctions, desmosomes, and hemidesmosomesare principally responsible for the mechanical and tensile strength of the barrier. Please note: This figure is not to scale.

The paracellular pathways are stringently regulated only to permit the passage of certain solutes and fluids, creating a selectively permeable barrier [18]. The junctional complexes, tight junctions (TJ), adherens junctions, and desmosomes with connections to the intracellular cytoskeleton, seal the paracellular space and provide structural support [18] (Figure 1).

Claudins are a family of proteins that regulate paracellular pathways across the intestinal epithelial barrier [19]. They form charge- and size-specific channels that allow permeation of solutes, water, and macromolecules through the TJs [19]. Occludin is a part of the tight junction associated marvel protein (TAMP) group along with tricellulin and marvelD3, with a role in cell polarity and TJ maintenance by interacting with other TJ proteins and intracellular actin and kinases [19,20]. Zonulaoccludens (ZO) are scaffolding membrane proteins that connect transcellular proteins to the intracellular cytoskeleton, therefore, have a role in assembly and maintenance of junctional proteins and paracellular permeability [21]. 

Adherens junctions (AJs) are basolateral to the TJs, and have important roles in cell-cell adhesion and signalling [9]. Various cadherin proteins interact to regulate the intracellular actin cytoskeleton and contribute to the formation of the perijunctionalactomyosin ring [18,22].

In conjunction with AJs, desmosomes provide the mechanical cohesion of intestinal epithelium, providing structural stability [17]. It is composed of various protein subunits including desmoglein, desmocolin, plakoglobin, plakophilin and desmoplakin. Its role in maintaining barrier function is largely unknown [23]. 

Many of these proteins can be aberrant in their abundances and thereby contribute to a weaker intestinal barrier [18]. Structural analysis of junction proteins revealed that there are fewer horizontal TJ strands and frequent strand discontinuities in IBD tissues, creating a paracellular route for macromolecule uptake [24]. The overall pattern of TJ protein abundances show consistent upregulation of pore-forming claudin-2 [10,24,25] (up to 10-fold increase in UC [26]) and down regulation of several barrier-enhancing claudins (claudin-3 [10,27], -4 [10,25,28], -5 [24], -8 [24]) as well as occluding [9,24,29,30,31] and ZO-1 [9,10,28,30] in IBD [32]. E-cadherin is the main component of the adherens junction and genetic polymorphism of this protein was identified to be associated with CD [32]. This highlights the role of junctional proteins and barrier function in the pathogenesis of IBD. Although decreased expression of colonic E-cadherin was associated with active disease [30], E-cadherin expression of the TI had no bearing on disease severity or intestinal barrier function [33].

## 3. Intracellular Regulators of Paracellular Permeability

Selective paracellular permeability is a critical component of a functional gastrointestinal (GI) tract, and is distinctive from other modalities of absorption in that no molecular transporters are involved and thus the rate and concentration of absorption is largely determined by transmural potential differences and concentration gradients [34]. In the healthy GI tract, passive paracellular chloride absorption facilitates a normal stool concentration in the range of 10–15 mmol/L. A number of critical nutrients and minerals are also passively absorbed through the paracellular pathway, and principally regulated by epithelial junctions to maintain homeostasis [34]. Some of these include oxalate, calcium, phosphate, and magnesium. Many of these molecules are also transcellularly absorbed, with the balance in transport modalities summarily dependent on the amount of the solute in the lumen (dietary intake) and physiological demand [34]. The paracellular luminal-tissue transport channel is largely regulated by the TJ and AJ complexes, and there is significant evidence of decreased expression and irregular distribution of TJ and AJ components including occludins, claudins and junctional adhesion molecules in IBD [35]. At the clinical level, IBD patients also appear to have higher rates of unregulated intestinal permeability via confocal endoscopic imaging [5]. TJ and AJ proteins are directly or indirectly (through protein-protein interaction) connected to the intracellular perijunctionalactomyosin ring [36]. Myosin Light Chain Kinase (MLCK) induces phosphorylation of myosin II regulatory light chain to cause contraction of the perijunctionalactomyosin ring, thereby, influencing the structure and function of the junctional proteins [37,38,39]. MLCK causes re-organisation of the perijunctional actin, occludin and ZO-1 [38], leading to the paracellular flux of uncharged macromolecules that is reversible with MLCK inhibition in experimental models [40]. There is an upregulation of MLCK in ileal biopsies of IBD patients, which correlates with disease activity [39]. However, MLCK appears to be an effector of inflammatory cytokines. Its expression is induced by Tumor Necrosis Factor (TNF) [41], and inhibiting MLCK can reverse barrier loss in the presence of TNFα [42]. This prevents TNF-α-induced caveolin-1-dependent occluding endocytosis [43,44], which is one of the predominant ways TNF-α causes barrier loss. 

Furthermore, an experimental model showed that increase in permeability of macromolecules from MLCK activation leads to an increase in IL-13 and subsequent claudin-2 expression; therefore, an increase cation permeability [40]. Hence, MLCK interacts with various inflammatory cytokines to modulate paracellular permeability. However, constitutively-active-MLCK mice showed mucosal immune activation (increased TNF-α, IFN-ϒ, IL-10, IL-13, and lamina propria T cells) but not spontaneous disease [45], suggesting that overactivation of MLCK alone is insufficient to cause IBD. The intracellular actin cytoskeleton itself can modify TJ function under the influence of various stimuli such as inflammatory cytokines, growth factors and microorganisms [22]. For example, Rho family of Guanosine Triphosphate hydrolase enzymes (GTPases) (a key molecule in intracellular actin signalling) can be inactivated by bacterial products (e.g., from *Clostridium difficile* and *Clostridium botulinum*), which results in re-organization of the F-actin in the perijunctionalactomyosin ring and alteration of TJ protein structures [44]. Other bacteria (e.g., *E. coli*) activate Rho GTPase to cause barrier loss via a different mechanism, sparing the perijunctionalactomyosin [22]. Also, depolymerisation of actin has been found to cause occludin re-distribution and internalisation via caveolae-mediated endocytosis which results in disruptions to the mucosal barrier [46]. In support of this finding, many studies have found TJ proteins in cytoplasmic vesicles following exposure to chemical and pathophysiological stimuli (e.g., calcium chelation, pathogenic *E. coli* infection, TNF) [22]. The Rho GTPasesignalling pathways have a complex interrelationship with MLCK that remains to be fully elucidated. Whilst both MLCK and Rho GTPase pathways phosphorylate MLC and appear to have complimentary roles in cell contractility and paracellular permeability, they seem to act at different sites of the cell—MLCK acts on the periphery of the cell to assemble microfilaments while Rho GTPases assemble stress fibres at the centre. MLCK is critical for maintaining basal stress fibres but does not affect late stress fibre reorganization [47]. Rho GTPases on the other hand, are critical in late stress fibre organization, and have been found to do so under TNF-α stress [47]. Rho GTPase subtype Cdc42 acts on PAK, a serine/threonine p21 activating kinase that phosphorylates MLCK and inactivates it, leading to tight junction disruption and intestinal barrier leak [43,48]. Whether MLCK and Rho GTPase MLC phosphorylation and Cdc42 induced phosphorylation of MLCK forms a part of a larger barrier function regulation loop remains an evolving subject.

## 4. Epithelial Restitution and Healing

The intestinal epithelial lining is continuously shed and replaced, maintaining the homeostasis between cell shedding and renewal [49]. Stem cells from the crypt differentiate and migrate to the villus tip in small bowel or colonic surface where they are shed. The dying cell signals to the surrounding cells to contract the actomyosin structure, which will extrude the dying cell out [50]. This is detected by a stretch-sensitive channel, which causes re-distribution of TJ proteins to transiently seal the gap left by the dying cell to maintain an intact barrier [50]. Similarly, the intestinal epithelium restores tissue integrity following any injury or damage in two steps: epithelial restitution (re-organisation of adjacent cells and TJs) and wound-healing (maturation and differentiation of stem cells and cell migration) [51]. These processes are critical in IBD as recurrent and extensive mucosal damage occur with disease activation. The mucosal biopsy of IBD expresses activated caspase-1 and -3 which is associated with intestinal barrier defects from a higher rate of epithelial cell extrusion [51]. Various cytokines and growth factors affect epithelial restitution and wound healing (Table 1). Other elements involved are goblet cells, immune cells (e.g., macrophages and T cells produce IL-6 and TNF, fibroblastsproduce hepatocyte growth factor to regulate epithelial cell regulation [52]), molecular pathways (e.g., canonical Wnt/β-catenin pathway in epithelial proliferation [2]), and the actin cytoskeleton and its regulators (e.g., Rho GTPase in epithelial restitution and toll-like-receptor function (TLR2 in synthesis of TTF3) [49,52].

Increased rates of cell shedding and subpar epithelial restitution and healing observed in patients with IBD may explain the higher number ofepithelial gaps and microerosions, potentially creating a route for the uptake of luminal content [53]. However, the role of microerosions or epithelial gaps on the barrier function is uncertain [53].

## 5. Clinical Implications of Impaired Intestinal Permeability in IBD

Barrier dysfunction is defined as loss of the continuous layer of the intestinal epithelium with interruptions in the interepithelial junctions and epithelial gaps [54]. This allows the permeation of microorganisms, dietary antigens and other noxious particles into the laminalpropria, resulting in the activation of the mucosal immune system and the inflammatory sequela of IBD [54]. The importance of intestinal barrier function in IBD has been recognised for decades [2,8,55]. However, it is still under debate whether the mechanisms that result in barrier loss are the primary cause of IBD or the consequence of a separate underlying pathology. 

Increased permeability appears to correlate better with symptoms than endoscopic activity [56] and predicts relapse better than other clinical and blood markers [6]. The emergence of a novel imaging technique, confocal laser endomicroscopy (CLE), sparked the comprehensive exploration of the functional and structural features of the intestinal barrier [57] (Table 2). These studies have consistently highlighted the pervasiveness of intestinal barrier dysfunction in the pathogenesis of IBD. Firstly, the features of impaired barrier function distinguish patients with IBD from healthy controls and these abnormalities persist in the absence of active clinical disease and affect the entire gastrointestinal tract [58,59,60]. The important features of impaired barrier function include fluorescein leakage, which is shown by an efflux of intravenous fluorescein contrast into the intestinal lumen and a loss of continuous epithelium (e.g., cell drop-out, epithelial gaps, or microerosions) [61]. Secondly, barrier loss has been found to be a reliable predictor of relapse and serious complications in IBD patients [13,60,62,63,64,65]. Within one year, an abnormal epithelial barrier is associated with an 80% risk of relapse and 45% risk of major events (such as hospitalisation or surgery), compared to 20% and 5% for those with normal epithelial barrier function [62]. Structural changes and increased intestinal permeability of the colon also accurately predicted relapse over a 12-month follow-up period of UC patients [63,64]. Similarly, a defective mucosal barrier of TI is associated with a significant risk of relapse in both UC and CD [13,60,65]. Lastly, some but not all features of barrier dysfunction may be reversed with treatment [33]. Karstensen et al. followed up CLE features of patients with UC in response to medical therapy and found that structural features such as crypt changes improved in response to medical therapy but not fluorescein leakage, a functional parameter delineating intestinal permeability [33]. This finding furthersupports the idea that intestinal barrier dysfunction may be a primary pathologic feature of IBD, and current immunosuppressive and anti-inflammatory therapies maynot restore complete tissue integrity. 

On the other hand, leaky gut has been observed in healthy relatives [12] and spouses of patients with CD [66,67] and some experimental and animal models with defects in various barrier components do not result in spontaneous inflammation [45]. Therefore, the causes of barrier dysfunction may be multifactorial and may not be the predominant pathogenic process in IBD. Some of the molecular changes in TJs and increased paracellular permeability observed in IBD patients are limited to patients with active disease and absent in remission. Such findings suggest that the increased permeability may be secondary to another inflammatory cause [24]. As yet, we can only comprehend with certainty that IBD is a complex disease with multiple contributing factors that interact with one another to result in the disease phenotype. The exact place for the loss of mucosal barrier function in the puzzle of IBD pathogenesis is obscure; however, the evidence indicates that barrier dysfunction predisposes or enhances disease progression in IBD. 

## 6. Assessing Barrier Function in Clinical Practice Today

Although the exact molecular pathogenesis behind barrier loss in IBD is uncertain, the intestinal epithelial barrier is an unequivocal source of important clinical information. However, the key obstacle in the assessment of intestinal barrier function in IBD is the lack of cost-effective and acceptable tools. The current methods for assessing intestinal barrier function and epithelial integrity have pronounced limitations, as outlined in Table 2.

In the past, sugar tests and Ussing chambers have been commonly used in research studies to study intestinal permeability. The gold standard has been lactulose/mannitol testing, in which the urinary excretion of a large sugar (lactulose), which generally does not cross the intestinal barrier, and a small sugar (mannitol), which freely crosses the intestinal barrier, are measured. Sugar tests require strict dietary restriction of sugars for 5–6 h, which is inconvenient for patients, and where test accuracy is heavily reliant on their compliance. Small sugars and other molecular probes such as polyethylene glycols (PEG 4000, 1500, 400) and radioactively labelled Cr-EDTA have numerous confounding factors such as intestinal motility, transit time, renal excretion, and bacterial degradation. These tools are unable to discern intestinal permeability at distinct sites (e.g., inflamed versus non-inflamed tissue). 

CLE with intravenous fluorescein contrast is a functional endoscopic imaging technique that allows 1000× magnification of the intestinal wall to visualise the epithelial lining and vasculature. It is recognised to be an excellent tool for the assessment of the barrier function as it can show structural and functional features of the intestinal epithelium [61]. In recent years, CLE has been used to study the intestinal barrier function of IBD patients [61]. The findings have accentuated the role of intestinal barrier function in the pathogenesis and natural history of IBD. Restoration of intestinal barrier function as assessed by CLE demonstrates cellular level evidence of remission and has been suggested as the new gold standard of mucosal healing [68]. 

One of the biggest limitations of CLE is the lack of a single standardised and validated system for interpreting the measurable parameters. With growing research using CLE, there is increasing heterogeneity in study protocols and interpretation of images reference required. Numerous researchers have devised scoring systems to evaluate intestinal barrier function using CLE. Watson’s grade is a categorical grading classification based on functional and structural abnormalities of the TI [13]. On CLE, fluorescein leakage (FL) and microerosions (defined as an epithelial gap with a diameter greater than one cell) are graded as Watson’s grade 2 and 3, respectively, and grade 1 defines normal barrier function. Watson’s grade has been validated against clinical outcomes and histology and replicated by several studies and applied to other areas of the GI tract (GIT) [13,58,59]. The sensitivity, specificity and accuracy of Watson’s score to predict for relapse in IBD is 62.5%, 91.2% and 79%, respectively [60].

The Confocal Leak Score (CLS) is a continuous scoring system [69]. It is calculated by the number of images showing three key features of leak proportional to the total number of images reviewed [69]. These features are fluorescein leakage, cell junction enhancement (the accumulation of fluorescein between epithelial cells, representing TJ abnormalities) and cell dropout or an epithelial gap [69]. CLS has a significant correlation to clinical symptoms in patients with mucosal healing (i.e., endoscopic remission). A CLS greater than 13.1 correlated to ongoing bowel symptoms, with every increase of CLS 1.9 associated with an extra diarrheal motion a day [5].

The Chang-Qing scale classifies colonic features of UC into four types based on the regularity of crypt arrangement, crypt density, dilation of crypt openings and crypt destruction and FL [63]. This has been validated against endoscopic and histologic assessment and clinical outcomes, with a sensitivity of 64%, specificity of 88.9% and accuracy of 74.4% at predicting relapse in UC [63].

The significance of some of these CLE features has been questioned by some studies. For instance, epithelial cell extrusion and gap have been found to be higher in patients with IBD in several studies, and this has been validated against histology and clinical outcomes [51,62]. By contrast, a study found that although there were more epithelial gaps in IBD patients compared to controls, this did not correlate with disease activity nor correlate to risk of hospitalisation or surgery [53]. Similarly, Kiesslich et al. found that only microerosions and not cell shedding have a prognostic significance in IBD [13]. Other controversial CLE features include vascular changes and the presence of inflammatory infiltrates [57].

The technical limitations of CLE have been listed in Table 2. Areas in need of further study include randomised control studies with standardized definitions of “barrier loss” and measures of disease activity [57], and direct comparison of CLE with conventional measures of intestinal permeability such as sugar tests and Ussing chambers. 

A few biomarkers of intestinal epithelial cell damage and inflammation have been discussed in the literature including plasma citrulline, fatty acid binding protein and faecal calprotectin [17]. However, these are markers of epithelial damage or inflammation rather than measures of intestinal permeability [17]. It is questionable whether subtle changes in barrier function that may precede active disease would be accurately reflected by these biomarkers. 

## 7. Biomarkers of Intestinal Barrier Function

Barrier function is affected by various factors and some of these have been shown to be defective or altered in IBD, prompting the invasion of pathogenic organisms. The differential expression of proteins that are directly involved in or regulate the interactions between the intestinal epithelium, the immune system and the intestinal microbiota have potential as biomarkers of intestinal barrier function in IBD.

A handful of studies have investigated the role of specific proteins in the regulation of intestinal permeability from human tissue samples (Table 3).Some affect the paracellular pathway by acting on the junctional proteins (e.g., Protein C pathway [73], prion protein (PrPc) [23], PECAM1) and the actin cytoskeleton (RTN-4B [74]), while others target epithelial homeostasis e.g., epithelial apoptosis (JAM-A). The studies included in this review focus on specific proteins previously known to contribute to the intestinal mucosal barrier, i.e., whole proteins have been quantified using immunohistochemistry rather than a rigorous quantitative proteomic approach that include quantification of peptide fragments. A global proteomic approach to biomarker discovery using mass spectrometry has not been done in this field before, which may lead to discovery of novel biomarkers. Current studies have found significant differences in protein expression, but only a few have been proposed as biomarkers of barrier function for several reasons: the differential protein analysis is limited to tissue (requiring endoscopy and biopsy for testing), insufficient difference in expression (<2-fold difference), and lack of validation data (no confirmatory studies due to time and cost involved). Furthermore, it is difficult to draw a conclusion as these studies have varying definitions of important parameters such as disease subtype, disease activity, response to treatment and control populations, with some studies including diseases known to involve altered intestinal permeability (e.g., IBS and Celiac disease) [54]. 

More importantly, none of these studies quantify these proteins against the parameter that measures the intestinal barrier function. This makes the results ambiguous and conflicting to discern whether the findings are attributed to the impaired barrier function seen in IBD patients or a result of their disease.

## 8. Conclusions

The current body of evidence demonstrates the importance of epithelial barrier function in the pathogenesis and natural history of IBD. However, there are profound limitations in current methods of assessing this feature in clinical practice, most of which revolve around the time-consuming and/or invasive nature of current techniques, that make them logistically unpractical in chronically ill patients. The discovery of biomarkers that can accurately assess intestinal barrier function and epithelial integrity would be a useful tool in predicting disease course and relapse, and assessing mucosal healing in IBD as well as other disorders associated with barrier dysfunction (e.g., Celiac disease and irritable bowel syndrome, amongst others) [54]. Many studies have explored specific proteins that contribute to the intestinal mucosal barrier. However, a proteomic study of IBD tissue in association with leaky gut for global identification and characterisation of proteins has not been done to date, which may lead to unexpected and novel discoveries of proteins that play vital roles in the intestine and have prognostic value in IBD. 

## Figures and Tables

**Table 1:** Regulatory factors of epithelial restitution and wound healing [49,52].

Action	Regulatory Factors
Inhibit cell proliferation	TGF-β
	Activin A
Promote epithelial restitution via TGF-β dependent pathway	Epidermal growth factor (EGF)
	Glucagon-like-peptide-2 (GLP-2)
	IL-1
	IFN-ϒ
	IL-2
	HGF
	VEGF
	FGF
Promote epithelial restitution via TGF-β independent pathway	Trefoil peptides
	Galectin-2
	Galectin-4
	Keratinocyte growth factor (KGF)
Decrease epithelial restitution velocity	IL-13 [32]
Promote epithelial proliferation	Epidermal growth factor (EGF)
	TGF-α
	IL-6IL-22
Induce cell apoptosis	TNF-α
Prevent cell apoptosis	Prostaglandin E2

**Table 2:** Assessment of intestinal permeability [17,54].

Technique	General Principle	Test Site	Test Method	Limitations
Molecular Probes				
Lactulose/mannitol	Oligosaccharides of different sizes	Small intestine	Urine	Time-consuming. Metabolised in the colon so limited application in assessing the large intestine (e.g., ulcerative colitis (UC)). Does not show permeation of bacterial components. Mannitol is contraindicated with blood transfusions.
Sucralose	Sucralose	Colon	Urine	Time-consuming. Does not show permeation of bacterial components.
Multi-sugar test	Sucrose, lactulose, sucralose, erythritol, rhamnase	Whole intestine	Urine	Time-consuming. Does not show permeation of bacterial components.
51Cr-EDTA	51Cr-EDTA crosses the intestinal barrier via the paracellular route and has similar physiological properties to oliogosaccharides.	Whole intestine	Urine	Invasive and complex detection method. Not readily available. Radioactivity. Impractical in clinical setting. Does not show permeation of bacterial components.
PEG4000/400	Polyethylene glycol, an inert molecule of different sizes.	Whole intestine	Urine	Time-consuming. The exact route of PEG is not well defined [70], thus implications in interpreting results. Does not show permeation of bacterial components.
Gadolinium-based MRI contrast agent [71]	Gadolinium (500–1000 Da)	Whole intestine	24-h urine collection	Lack of evidence in human studies. More expensive and may have higher toxicity than conventional sugars. Partial hepatobiliary elimination. Contraindicated in renal impairment.
Ussing chambers	Ion transport across the intestinal epithelium tissue sample is measured using a short circuit current.	Site-specific	Biopsy	Invasive and complex detection method. Ex-vivo. Lack of correlation between Ussing chamber and other permeability assays.
Imaging				
Confocal laser endomicroscopy	Intravenously-administered fluorescent contrast is seen to leak through the small intestinal mucosa under real time endoscopy.	Terminal ileum, colon, duodenum	Endoscopy	Invasive. Time-consuming (average of 46.5 minutes [60]). Validated measurement scores include the Watson grade (semi-quantitative [60]) and confocal leak score (quantitative) [5]. Requires special training of the endoscopist. Does not show permeation of bacterial components.
Biomarkers of Intestinal Permeability				
Claudin-3 [27]	Epithelial tight junction protein	NA	Urine	Limited data and lack of randomised trials.
Bacteria-Related Markers				
Lipopolysaccharide (LPS) assay	Show endotoxemia from bacterial translocation due to barrier function failure.	Colon	Blood (portal venous)	Technical limitation in detecting low levels of LPS in the peripheral blood. Requires careful standardization of the measurement. Evidence of use in Inflammatory Bowel Disease (IBD).
Circulating endotoxin core antibodies	An indirect measure of translocation of bacterial products by quantifying immunoglobulins (IgG, IgM and IgA) against the inner core of endotoxin for acute phase of intestinal barrier damage and function [72].	Colon	Blood	Only study done on post-operative patients, not patients with chronic gastrointestinal disease. Evidence for use in IBD.
Plasma d-lactate	d-lactate is produced by the gut bacteria and translocated across the intestinal mucosa with barrier dysfunction.	Colon	Blood	False positive test with bacterial over growth. Limited use in critically ill patients (e.g., ischemic colonic injury, acute necrotizing pancreatitis).
Faecal butyrate concentrations	Butyrate is a barrier enhancing substance, modifying claudin-1 and -2 to preserve intestinal barrier function and preventing bacterial translocation.	Colon	Faeces	Poorly established. The test relies on the principle that butyrate as a single major component of the barrier function rather than a complex and interactive entity.
Bacteria-derived haemolysin	Toxin that impair the intestinal barrier.	Colon		Poorly established. Results are attributed to only haemolysin-producing bacteria.
Assessment of fatty liver disease	Inflammation and fatty liver disease result from translocation of bacteria and its products into the portal system.	Whole intestine	Imaging	Poor specificity.

**Table 3:** Studies showing differential expressions of proteins contributing to the intestinal barrier function in IBD tissue.

Reference	Sample	Sample Size	Technique	Findings
Gassler et al., 2001 [9]	Surgical specimen	10 ulcerative colitis(UC) 10 Crohn’s disease(CD) 10 sporadic colon cancer	Reverse transcription Quantitative PCR and sequencing reaction Immunofluorescence staining and immunoblotting Immunohistochemistry Western blot and densitometric analysis	In actively inflamed Inflammatory Bowel Disease(IBD) tissue: desmosome protein expressions (desmoplakin-1, desmoglein-2 and desmocllin-2) decreased with severity of inflammation in IBD tissue (*p* < 0.05); Adherens junction(AJ) proteins such as E-cadherin and α-catenin were highly reduced; APC, p 120, plakophilin-2, β-catenin and plakoglobin were decreased and correlated with degree of inflammation in UC; plakophilin-2 and plakoglobin, but not β-catenin or APC proteins were reduced in actively inflamed CD; Tight junction(TJ) strands were discontinuous with reduced ZO-1 and occludin expression. In inactive IBD tissue: AJ-associated proteins were affected, but not desmosomes and TJs. Therefore, these alterations are not a primary occurrence in IBD.
Kucharzik et al., 2001 [30]	Colonic biopsy	11 active UC 9 active CD 29 control (normal colorectal mucosa or surgical resection of colon cancer)	Immunofluorescence Immunohistochemistry Western blotting	Global downregulation of occludin in IBD compared to controls. In epithelial cells adjacent to transmigrating polynorphonuclear leukocytes(PMNs), expressions of other TJ and AJ proteins were also downregulated (i.e.,zonulin-1 (ZO-1), claudin-1, junction adhesion molecule(JAM), beta-catenin, and E-cadherin).
Blair et al., 2006 [39]	Biopsy	5 UC 15 CD 6 control (adenocarcinoma)	Quantitative immunofluorescence microscopy	Epithelial MLCK expression mildly upregulated in inactive IBD and further upregulated in active disease (increase in Myosine Light Chain Kinase(MLCK) expression correlate with histological disease activity). MLCK phosphorylation is also significantly increased in active, but not inactive IBD.
Zeissig et al., 2007 [24]	Sigmoid colon biopsy	23 active CD 22 control 15 inactive CD 15 UC	Ussing chamber Freeze fracture electron microscopy Western blot Immunohistochemistry	Occludin (*p* < 0.05), claudin-5 (*p* < 0.05) and -8 (*p* < 0.001) were downregulated and re-distributed in active CD compared to controls but not in inactive state. Claudin-2 was strongly upregulated and inducible by Tumor Necrosis Factor- α (TNF-α). Other claudins were unchanged (-1, -4. -7) or not detectable in sigmoid colon (claudin-11, -12, -14, -15, and -16). There were reduced and discontinuous TJ strands. Focal epithelial lesions (e.g., microerosions) did not contribute to barrier dysfunction in CD. However, epithelial apoptosis was increased in active but not inactive CD.
Vetrano et al., 2008 [75]	Tissue specimen	11 control 13 CD 15 UC	Western blot Immunofluorescence staining for anti-JAM-A, E-cadherin and ZO-1 and confocal fluorescence microscopy	Loss of JAM-A expression in actively inflamed IBD (*p* < 0.01) but not in uninvolved mucosa of IBD. Western blot showed significantly lower JAM-A levels in inflamed mucosa of IBD (*p* < 0.05) compared to the controls.
Oshima et al., 2008 [25]	Rectum biopsy	5 active UC 5 control	Antibody staining (for claudin-1, 2, 3, 4, and 7) Immunofluorescence microscopy Western blot Real-time PCR	Expression of claudin-4 and -7 were decreased; claudin-2 was elevated and claudin-1 and -3 remained unchanged, compared to the control patients.
Thuijls et al., 2010 [27]	Colonic biopsy (only from IBD group) Urine samples	10 healthy 10 IBD remission (5 CD, 5 UC) 10 active IBD (4 CD, 6UC)	Immunostaining of claudin-3 Western blot for urinary claudin-3	Less staining of claudin-3 was observed in tissue samples of active IBD compared to controls and IBD patients in clinical remission. This correlated with urinary claudin-3 levels (*p* < 0.001).
Poritz et al., 2011 [29]	Mucosa sample	UC CD Control	Western blot	Decrease in occludin and an increase in claudin-1, thus significant increase in claudin-1: occludin (C:O) ratio in diseased UC colon compared to non-diseased UC colon (*p* < 0.001) and normal colon tissue (*p* < 0.01). In CD, C:O ratio elevated in all CD tissue, irrespective of disease status.
Vetrano et al., 2011 [73]	Colon biopsy	16 healthy 12 active CD 13 active UC	Immunohistochemistry Flow cytometry RT-PCR	EPCR (endothelial cell PC receptor) and PC (protein C) expression in inflamed tissue samples from UC and CD was significantly lower compared to healthy individuals (*p* < 0.001). EPCR, PC and PAP-1 (Protease-activated receptor-1) were expressed by epithelial cells of both healthy and IBD but the expression was decreased in IBD epithelial cells by 47% and 30%. Downregulation of mRNA for EPCR, PC as well as PAR-1 in active IBD.
Das et al., 2012 [28]	Colonic biopsy	11 active CD 10 active UC 10 untreated colonic tuberculosis 6 IBS as control	Immunohistochemistry Transmission electron microscopy	Claudin-2 upregulated in all disease groups (*p* = 0.002). Claudin-2 was expressed the full length of ICJ in IBD group while it was localised to the upper one-third in cTB and control groups. Claudin-4 expression was lower in disease compared to controls groups (*p* < 0.01). ZO-1 expression was reduced and focal in all disease group while it was diffused in control. Occludin expression were not significantly deviated in disease groups versus the control. Pentalaminar structure of TJ destroyed in IBD patients.
Petit et al., 2012 [23]	Colon samples from IBD patients	24 IBD patients Control (colonic diverticulitis)	Immunohistochemistry Electron microscopy Immunoblotting	PrPc was concentrated at cell-cell junction and largely co-localised with beta-catenin in controls. This was disorganised in the junctions of IBD mucosa, accompanied by an increase in intracellular signal. However, the mRNA and protein level of PrPc was not significantly deviated compared to the controls.
Goswami et al., 2014 [10]	Duodenal biopsy	24 Celiac disease 28 active CD 15 functional dyspepsia as controls	Light microscopy Immunohistochemistry Western blot Transmission electron microscopy	Overexpression of claudin-2 (*p* = 0.001 at villi and *p* = 0.007 at crypts) that did not reverse with six months of treatment. Occludin was significantly overexpressed (*p* < 0.001) compared to controls that did not decrease with treatment. ZO-1 was reduced in mucosal crypts (*p* = 0.004) that did not alter with treatment, however, western blotting did not find consistent results. No change in JAM-1 protein. Altered ultrastructure of TJs such as pentalaminarstructure and TJ dilatation.
Rodriguez-Feo et al., 2015 [74]	Tissue biopsy	15 inflamed CD 6 non-inflamed CD (control)	Immunohistochemistry, confocal microscopy, real-time PCR, Western blotting	IBD patient samples showed significant reduction of RTN-4B/NOGO-B expression in inflamed mucosa compared to non-inflamed mucosa which show patchy staining pattern mostly at surface epithelium.
Gu et al., 2017 [76]	Colon biopsy	40 IBD in remission (assessed at 6, 12, 24 months after baseline colonoscopy)	Quantitative real-time PCR Gene array	Baseline expression of platelet endothelial cell adhesion molecule (PECAM-1) (2.4 fold elevation, *p* = 0.02), ICAM-3 (1.9fold elevation, *p* = 0.03) and VCAM-1 (1.4fold elevation, *p* = 0.02) were significantly higher in patients who flared than those who did not. Elevation in PECAM-1 and ICAM-3 were significant as early as six months.
